# Incorporation of mutations in five genes in the revised International Prognostic Scoring System can improve risk stratification in the patients with myelodysplastic syndrome

**DOI:** 10.1038/s41408-018-0074-7

**Published:** 2018-04-04

**Authors:** Hsin-An Hou, Xavier Cheng-Hong Tsai, Chien-Chin Lin, Wen-Chien Chou, Yuan-Yeh Kuo, Chieh-Yu Liu, Mei-Hsuan Tseng, Yen-Ling Peng, Ming-Chih Liu, Chia-Wen Liu, Xiu-Wen Liao, Liang-In Lin, Ming Yao, Jih-Luh Tang, Hwei-Fang Tien

**Affiliations:** 1 Division of Hematology, Department of Internal Medicine, National Taiwan University Hospital, Taipei, Taiwan; 2 Tai-Cheng Stem Cell Therapy Center, National Taiwan University, Taipei, Taiwan; 3 Departments of Laboratory Medicine, National Taiwan University Hospital, Taipei, Taiwan; 4 Graduate Institute of Oncology, College of Medicine, National Taiwan University, Taipei, Taiwan; 5 Biostatistics Consulting Laboratory, Department of Nursing, National Taipei College of Nursing, Taipei, Taiwan; 6 Department of Pathology, National Taiwan University Hospital, Taipei, Taiwan; 7 Department of Clinical Laboratory Sciences and Medical Biotechnology, College of Medicine, National Taiwan University, Taipei, Taiwan

**Keywords:** Cancer genetics, Myelodysplastic syndrome

## Abstract

Gene mutations have not yet been included in the 2016 WHO classification and revised International Prognostic Scoring System (IPSS-R), which are now widely utilized to discriminate myelodysplastic syndrome (MDS) patients regarding risk of leukemia evolution and overall survival (OS). In this study, we aimed to investigate whether integration of gene mutations with other risk factors could further improve the stratification of MDS patients. Mutational analyses of 25 genes relevant to myeloid malignancies in 426 primary MDS patients showed that mutations of *CBL, IDH2, ASXL1*, *DNMT3A*, and *TP53* were independently associated with shorter survival. Patients within each IPSS-R or 2016 WHO classification-defined risk group could be stratified into two risk subgroups based on the mutational status of these five genes; patients with these poor-risk mutations had an OS shorter than others in the same risk group, but similar to those with the next higher risk category. A scoring system incorporating age, IPSS-R and five poor-risk mutations could divide the MDS patients into four risk groups (*P* < 0.001 for both OS and leukemia-free survival). In conclusion, integration of gene mutations in current IPSS-R improves the prognostication of MDS patients and may help identify high-risk patients for more aggressive treatment in IPSS-R lower risk group.

## Introduction

The myelodysplastic syndrome (MDS) is a heterogeneous group of clonal hematopoietic disorders characterized by various clinical presentations, risks for leukemia transformation and reduced survival^1,2^. This makes the accurate diagnosis, classification, and risk stratification of MDS patients clinically relevant^3^. A number of prognostic models, including International Prognostic Scoring System (IPSS), World Health Organization (WHO) based Prognostic Scoring System (WPSS), and the revised IPSS (IPSS-R), etc. have been developed to help risk-stratify these patients and facilitate clinical decision-making^4–6^.

Over the past decade, high throughput genetic techniques have uncovered novel genetic alterations and illuminated the genomic landscape of MDS^7–10^. Importantly, some of these mutations are associated with distinct clinical features and can predict survival^11–14^. Nevertheless, molecular data are not included in current prognostic systems. The 2001 WHO Classification included cytogenetics in MDS classification, and the updated 2016 WHO classification first includes the status of *SF3B1* mutation in the classification of MDS with ring sideroblasts^15,16^.

Recently, Haferlach et al.^12^ and Nazha et al.^17^ demonstrated that integrating mutational profiling into the IPSS-R could better discriminate MDS patients into various prognostic groups. However, the number of incorporated genes, patient population and treatment strategies were highly variable in these two studies. More investigations in the use of mutational data for MDS prognostication may give clearer view in this regard. Further, since the demographics and disease natures in MDS patients are different between Asian and Western countries^18–20^, it is unclear whether this new risk model combining both clinical parameters and genetic data are similarly useful for risk stratification of MDS patients in Asia. Besides, it remains to be answered whether the mutational status could further help improve the prognostic prediction in the 2016 WHO classification. In this study, we aimed to assess the prognostic impact of integration of gene mutations with conventional risk factors in a large cohort of patients with primary MDS in Taiwan. We found that patients within each IPSS-R or 2016 WHO classification-defined risk group could be stratified into two risk subgroups with different outcomes based on the mutational status of *CBL, IDH2, ASXL1*, *DNMT3A*, and *TP53*. Patients with these poor-risk mutations had an OS worse than others in the same risk group, but similar to those in the next higher risk group. Mutations screening in these five genes may help identify high-risk patients in IPSS-R lower risk group for more aggressive treatment.

## Patients and methods

### Patient cohorts

From 1984 to 2010, 426 adult patients with primary MDS who were diagnosed at the National Taiwan University Hospital (NTUH) and had complete clinical, mutational and cytogenetic data were included. Patients with secondary or therapy-related MDS were excluded to make the cohort more homogeneous. The diagnosis and classification of MDS were based on the French–American–British (FAB), 2008 and 2016 WHO criteria^15,16,21^. Among them, the disease of 328 patients fulfilled the criteria of MDS according to the 2016 WHO classification. Most of the patients received conservative and supportive care. Twenty-two patients (5.2%) were treated with acute myeloid leukemia (AML)-directed chemotherapy, 24 (5.6%) patients received hypomethylating agents (HMA), and 46 (10.8%) patients, allogeneic hematopoietic stem cell transplantation (HSCT). This study was approved by the institutional review board of the NTUH (20150709RINA) and written informed consent was obtained from all participants in accordance with the Declaration of Helsinki.

### Cytogenetics

Bone marrow cells were collected directly or after 1–3 d of unstimulated culture as described previously^22^. Metaphase chromosomes were banded by trypsin-Giemsa technique and karyotyped according to the International System for Human Cytogenetic Nomenclature.

### Mutation analysis

DNA was extracted from BM cells obtained at the time of diagnosis. Mutational analyses of 25 relevant genes involving in activated signaling pathways, such as *FLT3*-ITD^22^, *NRAS*^22^, *KRAS*^22^, *JAK2*^22^, and *CBL*;^23^ the transcription factors, such as *RUNX1*^24^ and *GATA2*;^25^ splicing factors, including *SRSF2*^26^, *U2AF1*^26^, *SF3B1*^26^, and *ZRSR2*;^27^ epigenetic modifications, including *IDH1*^28^, *IDH2*^28^, *DNMT3A*^29^, *TET2*^30^, *MLL/*PTD^31^, *ASXL1*^32^ and *EZH2*;^33^ *cohesin*genes^34^, including *RAD21*, *STAG1*, *STAG2*, *SMC1A*, and *SMC3*, as well as *SETBP1*^35^ and *TP53*^36^, were performed as previously described. Abnormal sequencing results were confirmed by at least two repeated analyses. To detect these mutations at diagnosis, we used DNA amplified in vitro from patients’ bone marrow cells by Illustra^TM^ GenomiPhi V2 DNA amplification kit as described by the manufacturer (GE Healthcare, Buckinghamshire, UK). All the mutations detected in such samples were verified in the original non-amplified samples.

### Statistical analysis

The discrete variables of patients with and without poor-risk gene mutations were compared using the *χ*^2^-tests, but if the expected values of contingency tables were smaller than 5, Fisher exact test was used. Mann–Whitney test method was used to compare continuous variables and medians of distributions. Overall survival (OS) was measured from the date of first diagnosis to the date of last follow-up or death from any cause. Leukemia**-**free survival (LFS) was defined as the time from the date of MDS diagnosis to the date of AML evolution or death from any cause, whichever came first. Kaplan–Meier estimation was used to plot survival curves, and log-rank tests were used to calculate the difference of OS and LFS between groups. Multivariate Cox proportional hazard regression analysis was used to investigate independent prognostic factors for OS. The proportional hazards assumption (constant hazards assumption) was examined by using time-dependent covariate Cox regression before conducting multivariate Cox proportional hazard regression. The variables, including age, gender, IPSS-R, and *CBL*, *RUNX1*, *IDH2*, *DNMT3A*, *TET2*, *ASXL1*, *EZH2*, *SRSF2*, *ZRSR2*, *TP53*, and *cohesin* gene mutations were used as covariates. A *P*-value <0.05 was considered statistically significant. All statistical analyses were performed with SPSS 18 software (SPSS Inc., Chicago, IL, USA) and Statsdirect (Cheshire, England, UK).

## Results

Supplementary Table 1 summarized the baseline characteristics of all enrolled patients. The median age at diagnosed was 67 years (range 18–98), with 58.9% patients older than 65 years. According to the FAB classification, 155 patients (36.4%) had refractory anemia (RA), 32 (7.5%) had RA with ring sideroblasts (RARS), 141 (33.1%) had RA with excess blasts (RAEB), 46 (10.8 %) had RAEB in transformation (RAEBT), and 52 (12.2 %), chronic myelomonocytic leukemia (CMML). In the 328 patients with MDS defined by the 2016 WHO classification, most had MDS with multilineage dysplasia (MDS-MLD, 25.3%), followed by EB2 (22.3%), EB1 (20.7%) and MDS with single-lineage dysplasia (MDS-SLD, 20.1%). By IPSS-R, 27.7% of the entire cohort had lower risk (very low and low risk), 25.3% had intermediate risk and 47% had high-risk MDS (high and very high risk).

### Frequency and distribution of gene mutations

In mutational analyses of 25 genes, 285 (66.9%) of 426 patients harbored at least one molecular genetic alteration; whereas, cytogenetic studies identified karyotypic abnormalities in 37%. Taken together, 77.4% patients had either gene mutation or cytogenetic change at diagnosis. One hundred and 82 (42.7%) patients had two or more mutations. Unsurprisingly, the patients with advanced MDS, defined by the FAB, 2016 WHO classification or IPSS-R had more mutations compared with those with lower risk subtypes (*P* < 0.001; Supplementary Figure 1).

The frequent mutations with an incidence of >10% were *ASXL1* (22.5%), *TET2* (13.8%), *SRSF2* (13.6%), *RUNX1* (12.2%), and *SF3B1* mutations (11%, Table 1 and Supplementary Figure 2). Less common mutations included *DNMT3A* (9.9%), *ZRSR2* (9.6%), *TP53* (9.6%), *U2AF1* (7.3%), *STAG2* (6.3%), and *EZH2* (6.3%) mutations. Except *SF3B1* and *TET2* mutations, most of other mutations occurred more frequently in IPSS-R high- or very high-risk subgroup than in lower risk subgroups (Supplementary Figure 3). Together, the most common functional pathway involved in gene mutations in this MDS cohort was RNA splicing (39%), followed by DNA methylation (24.4%), chromatin modification (24.2%), and transcription (14.3%, Table 1 and Supplementary Figure 4).

**Table 1:** Mutation frequencies of 25 myeloid genes

Variables	No. examined	Mutated no.	Percentage (%)
*RAS* signaling pathway	426	37	8.7
* NRAS*	426	19	4.5
* KRAS*	426	6	1.4
* CBL*	426	13	3.1
Receptors/kinases	426	9	2.1
* FLT3/*ITD	426	5	1.2
* JAK2*	426	4	0.9
Transcription	426	61	14.3
* RUNX1*	426	52	12.2
* GATA2*	426	11	2.6
DNA methylation	426	104	24.4
* IDH1*	426	4	0.9
* IDH2*	426	18	4.2
* DNMT3A*	426	42	9.9
* TET2*	426	59	13.8
Chromatin modification	426	103	24.2
* ASXL1*	426	96	22.5
* EZH2*	426	27	6.3
* MLL*/PTD	426	5	1.2
RNA splicing	426	166	39
* U2AF1*	426	31	7.3
* SRSF2*	426	58	13.6
* SF3B1*	426	47	11.0
* ZRSR2*	426	41	9.6
Cohesin	426	31	7.3
* RAD21*	426	0	0
* STAG1*	426	2	0.5
* STAG2*	426	27	6.3
* SMC1A*	426	2	0.5
* SMC3*	426	0	0
Others	426	55	12.9
* SETBP1*	426	14	3.3
* TP53*	426	41	9.6

**Fig. 1:**
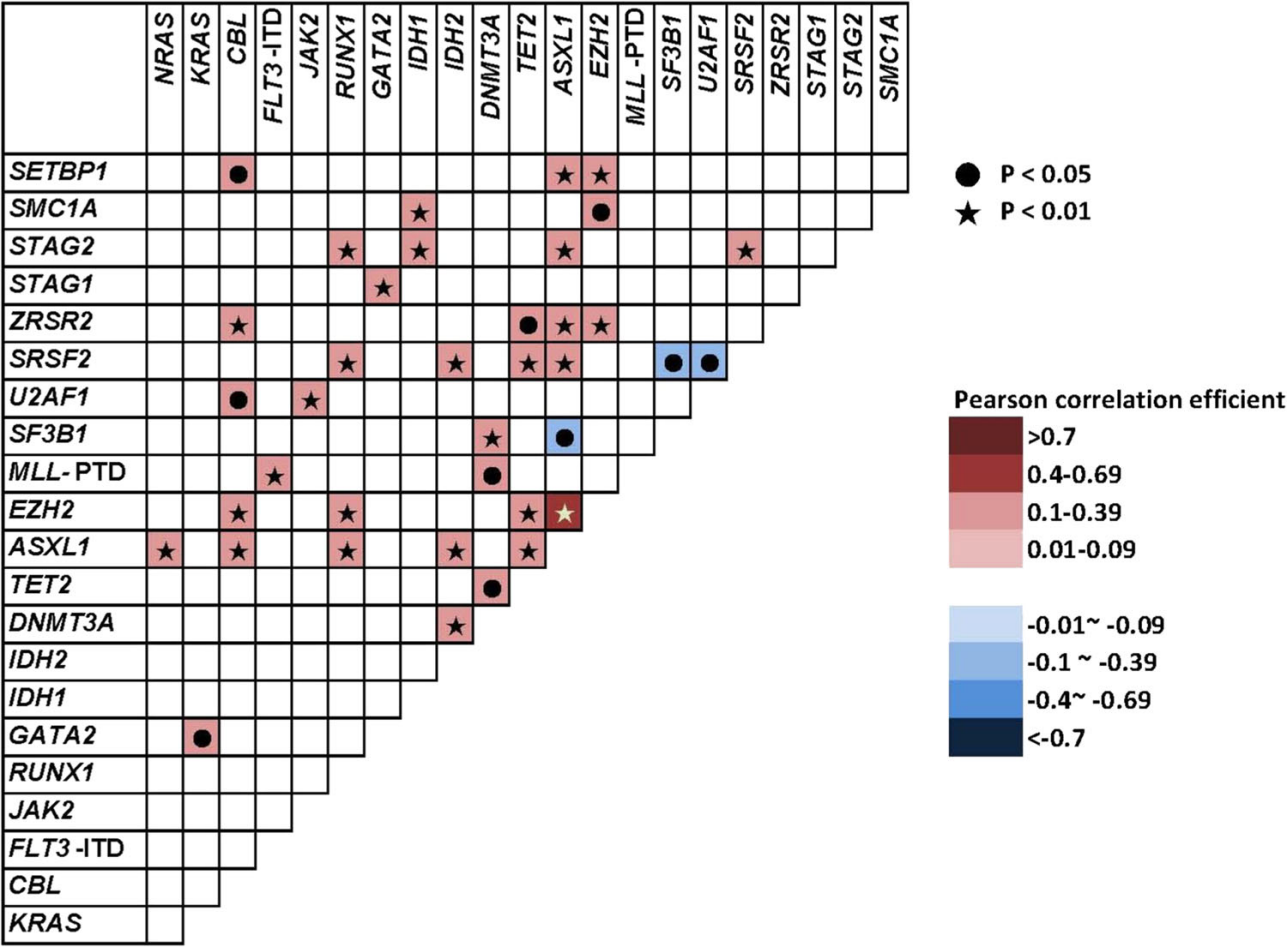
Pairwise associations among gene mutations in 426 MDS patients

Statistically significant positive or negative correlations were identified across 38 combinations of mutations, indicating the presence of co-operativeness and/or mutual exclusiveness of mutations in the pathogenesis of MDS (Fig. 1 and Supplementary Figure 5). In brief, mutations of genes involved in RNA splicing machinery or cohesin complex were mutually exclusive. On the other hand, *ASXL1* mutation, the most prevalent mutation in our sample, frequently coexisted with other mutations; it significantly co-mutated with *EZH2*, *STAG2*, *SRSF2*, *ZRSR2*, *TET2, IDH2, RUNX1*, and *SETBP1* mutations, but was exclusive with *SF3B1* mutation. *TET2* mutation was positively associated with *EZH2*, *ASXL1*, *SRSF2*, and *ZRSR2* mutations, *RUNX1* mutation with *EZH2*, *ASXL1*, *SRSF2*, and *STAG2* mutations, and *SRSF2* mutation with *ASXL1*, *RUNX1, IDH2, TET2*, and *STAG2* mutations.

### Prognostic impact of mutations

With a median follow-up of 43.2 months (range 0.1–250.7), the median OS for total cohort was 30.9 months. Because only 12 patients were of IPSS-R very low risk, we compiled the patients with IPSS-R very low and low risk together into survival analysis. Both OS and leukemia transformation rate were significantly different among the four subgroups of patients stratified by IPSS-R (both *P*-value <0.001; Supplementary Figure 6). We found that the clinical outcome got worse progressively as the patients had increasing number of somatic mutations (LFS and OS, *P* < 0.001; Supplementary Figure 7).

**Fig. 2:**
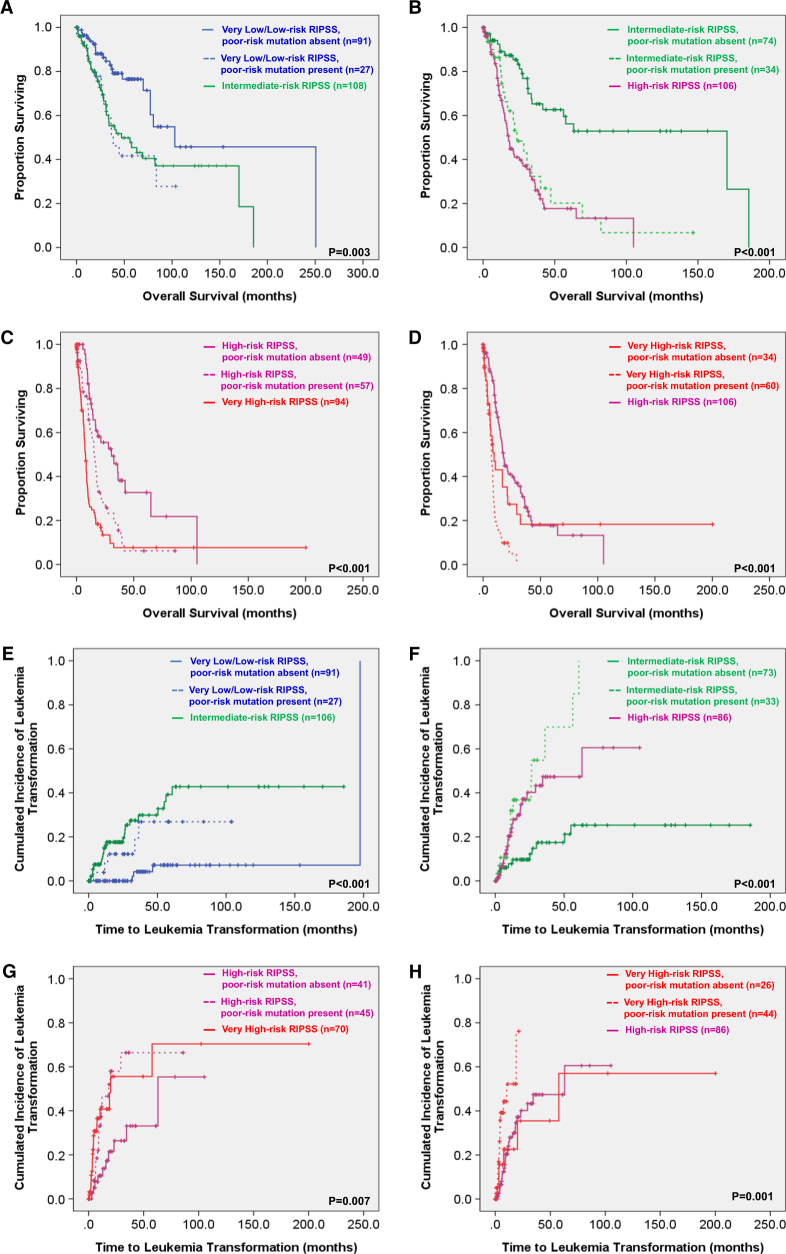
Overall survival and leukemia transformation rate according to the revised International Prognostic Scoring System (IPSS-R) risk categories and mutational status. **a**–**d** Overall survival according to the presence or absence of poor-risk mutations (*CBL, IDH2, ASXL1*, *DNMT3A*, and *TP53* mutations) and IPSS-R. **e**–**h** Leukemia transformation rate according to the presence or absence of poor-risk mutations (*CBL, IDH2, ASXL1*, *DNMT3A*, and *TP53* mutations) and IPSS-R

In univariate analysis, older age, male gender, and IPSS-R scores predicted prognosis in MDS patients. Besides, mutations in eleven genes, including *CBL*, *RUNX1*, *IDH2*, *DNMT3A*, *TET2*, *ASXL1*, *EZH2*, *SRSF2*, *ZRSR2*, *Cohesin*, and *TP53* were significantly associated with reduced OS (Table 2). In the multivariate Cox proportional hazards regression analysis, older age and mutations in *CBL*, *IDH2*, *DNMT3A*, *ASXL1*, and *TP53* were independent poor prognostic factors for OS, while lower IPSS-R scores had good prognostic impact (Table 3). Based on the above findings, we defined the five prognostic genes (*CBL*, *IDH2*, *DNMT3A*, *ASXL1*, and *TP53*) as poor-risk mutations. Totally, 178 (41.8%) patients harbored at least one poor-risk mutation. Compared with patients without poor-risk mutation, these patients were older, had higher WBC counts at diagnosis (Supplementary Table 2), higher risk of leukemia transformation (5-year leukemia transformation rate 61.7 ± 7.1% vs. 22.1 ± 4.1%, *P* < 0.001, Supplementary Figure 8) and shorter OS (median 15 months vs. 69.9 months, *P* < 0.001). Interestingly, presence of poor-risk mutations could further stratify the IPSS-R low or very low-risk patients into two groups with distinct outcomes (median OS, 38.4 months for patients with poor-risk mutations vs. 102.8 months for those without, *P* = 0.003; Fig. 2a). The survival of the IPSS-R very low or low-risk patients who harbored poor-risk mutation was similar to the IPSS-R intermediate-risk patients. Similarly, there were distinct intra-group differences in OS based on the presence or absence of poor-risk mutations among patients with IPSS-R intermediate, high, and very high-risk MDS (Fig. 2b–d). The same was also true for 5-year leukemia transformation rate (Fig. 2e–h). In other words, patients in each IPSS-R risk subgroup who harbored these unfavorable mutations had OS worse than other patients of the same risk but similar to those in the next higher risk subgroup. Although the poor-risk mutations had significant impact on clinical outcomes in all IPSS-R risk groups, it was much obvious in lower risk population. Intriguingly, the molecular data could further reclassify 22.9% (27/118) of IPSS-R very low/low risk patients to intermediate risk subgroup, 31.5% (34/108) of IPSS-R intermediate to high-risk subgroup, and 53.8% (57/106) of IPSS-R high to very high-risk subgroup.

**Table 2:** Univariate analysis for the overall survival and 5-year leukemia-free survival in MDS patients

Variable	No. of patients	Median OS ± SE, month	*P*-value	No. of patients	5-year LFS (%)	*P*-value
Age			0.001			0.556
≧65 years	221	26.3 ± 3.6		199	68.1 ± 6.1	
<65 years	205	39.7 ± 16.5		181	60.4 ± 5.2	
Gender			0.014			0.173
Male	281	26.3 ± 3.1		252	59.1 ± 5.1	
Female	145	41.6 ± 16.9		128	71.7 ± 5.8	
*IPSS*			<0.001			<0.001
Low/Int1	249	69.9 ± 12.4		249	73.5 ± 4	
Int2/high	177	10.5 ± 1.1		131	38.7 ± 10.4	
*IPSS-R*			<0.001			<0.001
Very low/low	118	83.6 ± 15.2		118	88.2 ± 4.1	
Intermediate	108	47.2 ± 14.6		106	60.8 ± 7.1	
High	106	17.7 ± 1.2		86	52.6 ± 7.8	
Very high	94	7.8 ± 0.6		70	29.6 ± 14.1	
*Mutations*						
*NRAS*			0.114			0.001
Mutated	19	31.9 ± 2.6		11	31.8 ± 17.5	
Wild type	407	15.9 ± 3.6		369	66.3 ± 4.1	
*KRAS*			0.524			0.302
Mutated	6	17.7 ± 4.6		5	53.3 ± 24.8	
Wild type	420	31.1 ± 2.8		375	65.5 ± 3.7	
*CBL*			0.001			0.009
Mutated	13	11.8 ± 2.1		12	38.6 ± 17.3	
Wild type	413	32.5 ± 2.7		368	66.1 ± 3.8	
*FLT3/ITD*			0.289			0.003
Mutated	5	16.9 ± 5.7		4	25.0 ± 21.7	
Wild type	421	31.3 ± 2.6		376	65.6 ± 3.8	
*JAK2*			0.989			0.396
Mutated	4	19 ± 7.0		3	100	
Wild type	422	30.9 ± 2.8		373	64.9 ± 3.7	
*RUNX1*			0.008			<0.001
Mutated	52	18.6 ± 4.0			34.6 ± 10.4	
Wild type	374	32.5 ± 2.4			68.8 ± 3.9	
*GATA2*			0.150			0.903
Mutated	11	NA		9	65.6 ± 20.9	
Wild type	415	30.9 ± 2.8		371	65.1 ± 3.8	
*IDH1*			0.285			0.402
Mutated	4	15 ± 3.5		2	0	
Wild type	422	31.9 ± 2.5		378	65.4 ± 3.7	
*IDH2*			0.02			0.081
Mutated	18	18.5 ± 6.6		13	56 ± 17.1	
Wild type	408	32.6 ± 2.5		367	65.9 ± 3.8	
*DNMT3A*			0.035			0.137
Mutated	42	16.9 ± 3.9		37	33 ± 23.8	
Wild type	384	32.7 ± 2.8		343	65.3 ± 4.0	
*TET2*			0.022			0.305
Mutated	59	20.1 ± 3.2		57	60.2 ± 10.1	
Wild type	367	31.9 ± 2.9		323	65.8 ± 4.0	
*ASXL1*			<0.001			<0.001
Mutated	96	18.7 ± 2.6		84	30.8 ± 9.7	
Wild type	330	36.3 ± 8.1		296	73.2 ± 3.8	
*EZH2*			0.044			<0.001
Mutated	27	17 ± 2.2		25	36.4 ± 14.1	
Wild type	399	32.5 ± 2.7		355	67.1 ± 3.8	
*MLL/PTD*			0.149			0.018
Mutated	5	16.9 ± 0.6		3	0	
Wild type	421	31.1 ± 2.8		377	66.7 ± 3.8	
*U2AF1*			0.316			0.1
Mutated	31	31.3 ± 4.9		27	13.9 ± 12.4	
Wild type	395	30.9 ± 3.1		353	69.5 ± 3.4	
*SRSF2*			0.001			0.014
Mutated	58	18.5 ± 3.7		52	24.6 ± 18.5	
Wild type	368	33.8 ± 3.3		328	68.3 ± 3.8	
*SF3B1*			0.482			0.645
Mutated	47	39.7 ± 11.3		43	70.6 ± 9.8	
Wild type	379	29.3 ± 3.1		337	64.4 ± 4	
*ZRSR2*			0.008			0.525
Mutated	41	14 ± 2.1		37	63.7 ± 11	
Wild type	385	32.7 ± 2.9		343	65.2 ± 3.9	
*SETBP1*			0.988			0.163
Mutated	14	36.4 ± 16.8		13	45.5 ± 16.6	
Wild type	412	31.1 ± 2.7		367	65.7 ± 3.8	
*TP53*			<0.001			<0.001
Mutated	41	6.6 ± 1.2		29	41.3 ± 11.9	
Wild type	285	36.1 ± 3.0		351	67.2 ± 3.8	
*Cohesin* ^a^			0.005			<0.001
Mutated	31	22.5 ± 4.3		23	21.5 ± 12.2	
Wild type	395	33.8 ± 3.4		357	68.1 ± 3.8	

*OS* overall survival, *CI* confidence interval, *IPSS* international prognostic scoring system, *IPSS-R* revised IPSS, *LFS* leukemia-free survival

^a^*Cohesin* genes, including *RAD21*, *STAG1*, *STAG2*, *SMC1A*, and *SMC3*

**Table 3:** Multivariate analysis (Cox regression) for the overall survival and leukemia-free survival in MDS patients

Variables	Overall survival				Leukemia-free survival			
		95% CI				95% CI		
	RR	Lower	Upper	*P*-value	RR	Lower	Upper	*P*-value
Age	1.024	1.014	1.034	<0.001*	0.997	0.983	1.011	0.713
Gender (male vs. female)	1.248	0.922	1.687	0.151	1.251	0.753	2.079	0.387
IPSS-R scores^a^	0.306	0.208	0.450	<0.001*	0.211	0.102	0.437	<0.001*
*CBL* mutation	2.292	1.164	4.513	0.016*	2.561	0.943	6.955	0.065
*RUNX1* mutation	1.021	0.679	1.536	0.920	1.381	0.729	2.617	0.322
*IDH2* mutation	1.957	1.045	3.665	0.036*	1.798	0.577	5.602	0.311
*DNMT3A* mutation	1.571	1.028	2.401	0.037*	1.789	0.875	3.658	0.111
*TET2* mutation	1.233	0.829	1.835	0.301	1.270	0.642	2.513	0.493
*ASXL1* mutation	1.557	1.040	2.329	0.031*	2.009	1.066	3.788	0.031*
*EZH2* mutation	1.292	0.724	2.304	0.385	1.413	0.587	3.402	0.440
*SRSF2* mutation	1.084	0.684	1.719	0.731	1.168	0.530	2.576	0.700
*ZRSR2* mutation	1.212	0.782	1.879	0.389	1.035	0.477	2.249	0.930
*Cohesin* mutation^b^	1.232	0.764	1.986	0.392	2.620	1.281	5.359	0.008*
*TP53* mutation	9.524	6.067	14.950	<0.001*	14.669	6.664	32.288	<0.001*

*RR* relative risk, *CI* confidence interval, *IPSS-R* revised international prognostic scoring system

*P-value <0.05 was considered significant

^a^IPSS-R scores: lower IPSS-R scores (very low- and low risk) vs. others

^b^*Cohesin* genes, including *RAD21*, *STAG1*, *STAG2*, *SMC1A*, and *SMC3*A

**Fig. 3:**
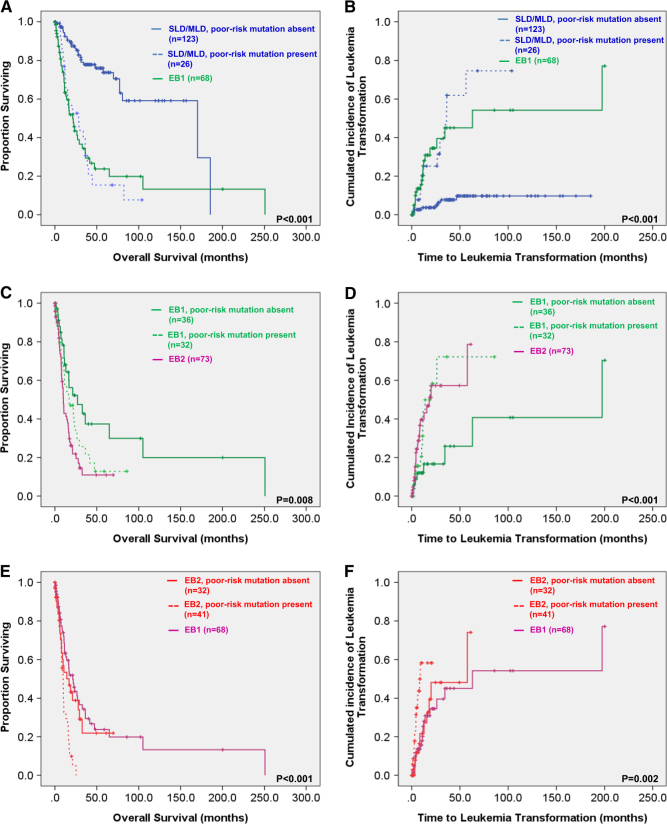
Overall survival and leukemia transformation rate according to 2016 WHO classification risk categories and mutational status. **a**, **c**, **e** Overall survival according to the presence or absence of poor-risk mutations (*CBL, IDH2, ASXL1*, *DNMT3A*, and *TP53* mutations) and 2016 WHO classification. **b**, **d**, **f** Leukemia transformation rate according to the presence or absence of poor-risk mutations (*CBL, IDH2, ASXL1*, *DNMT3A*, and *TP53* mutations) and 2016 WHO classification

Next, we investigated the impact of molecular data on clinical outcomes in MDS patients according to the 2016 WHO classification. We found the distinct survival difference of MDS patients stratified by the 2016 WHO classification (Supplementary Figure 9). The median OS was 170.2, 57.6, 21.3, and 10.1 months for MDS-SLD, MDS-MLD, EB1, and EB2, respectively (*P* < 0.001). The 5-year leukemia transformation rate was 17.2%, 24%, 45.1%, and 78.6%, respectively, for these four subgroups. Intriguingly, in the cohort of patients with MDS-SLD or MDS-MLD, poor-risk mutations could further stratify these patients into two risk groups; the patients with unfavorable mutations had higher probability of leukemia transformation and poorer OS than those without in the same subgroup, but comparable to those with MDS-EB1 (Fig. 3a, b). Similarly, in the cohort of patients with MDS-EB1, those with poor-risk mutations had worse clinical outcomes regarding both leukemia transformation and OS, but comparable to the next higher risk subgroup with MDS-EB2 (Fig. 3c, d). The same was also true for MDS-EB2 patients (Fig. 3e, f). However, presence of poor-risk mutations had no statistically significant impact on prognosis in the 34 patients with MDS-RS-SLD or MDS-RS-MLD, possibly due to limited number of patients in this group.

**Fig. 4:**
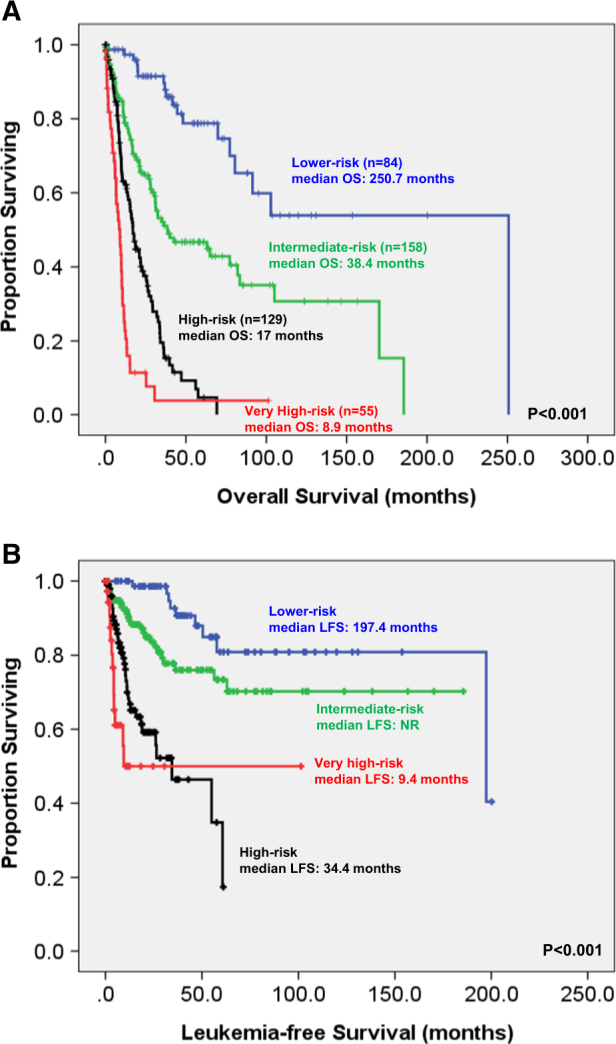
Kaplan–Meier estimates of overall survival (**a**) and leukemia transformation rate (**b**) based on integrated IPSS-R and mutational analyses in the 426 FAB-defined MDS patients

**Fig. 5:**
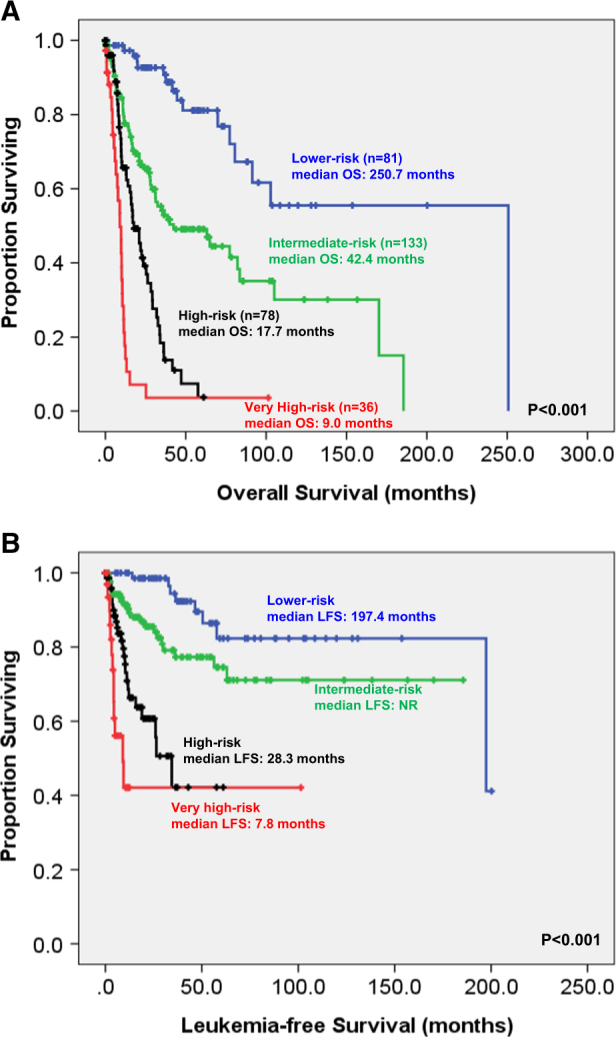
Kaplan–Meier estimates of overall survival (**a**) and leukemia transformation rate (**b**) based on integrated IPSS-R and mutational analyses in the 328 WHO-defined MDS patients

To better stratify the MDS patients into different risk groups, an integrated scoring system incorporating seven prognostic markers, including age, IPSS-R, and mutations of *CBL, IDH2, DNMT3A*, *ASXL1*, and *TP53*, into survival analysis was formulated. A new risk model was developed incorporating the weighted coefficients of these factors: age × 0.025—IPSS-R lower risk group × 1.184 + *CBL* × 0.829 + *IDH2* × 0.829 + *DNMT3A* × 0.452 + *ASXL1* × 0.442 + *TP53* × 2.254. Four risk groups were proposed: low (score <−0.5; *n* = 84), intermediate (score −0.5 ~ 0.5; *n* = 158), high (score 0.51 ~ 1.5; *n* = 129) and very high (score >1.5; *n* = 55). The median OS was 250.7, 38.4, 17, and 8.9 months for low, intermediate, high, and very high subgroups, respectively. This clinically relevant integrated scoring system divided the MDS patients into four groups with different clinical outcomes (*P* < 0.001 for both OS and LFS; Fig. 4). The same was also true for the 326 patients based on the 2016 WHO classification (*P* < 0.001 for both OS and LFS; Fig. 5).

Finally, since some patients in our cohort received allogeneic HSCT, we evaluated the impact of the treatment on OS in individuals with poor-risk mutations. The median OS was significantly longer in the patients receiving allogeneic HSCT than those without in this high-risk group (91.4 months vs. 13.7 months, *P* = 0.01; Supplementary Figure 10). In the cohort of patients with IPSS-R lower risk but unfavorable molecular genotype, the OS was similar to those without poor-risk mutation if they received allogeneic HSCT (*P* = 0.452). Similar finding was also found in IPSS-R higher risk cohort (*P* = 0.4902).

## Discussion

To our knowledge, there have been few studies integrating molecular data into IPSS-R in MDS patients^12,17,37^. Herein, we comprehensively analysed clinical parameters and 25 genetic alterations in a large series of MDS patients and found incorporation of mutational profiles into current IPSS-R and 2016 WHO classification may improve the prognostic stratification of MDS patients.

Because, the median survival and risk of leukemia transformation vary widely in the MDS patients, even in the same IPSS or IPSS-R group, it is prudent to identify novel prognostic markers to make the prediction more accurate. Bejar et al.^8^ first proposed a prognostic model using both IPSS and the mutation status of *RUNX1*, *ETV6*, *EZH2*, *ASXL1*, and *TP53* to provide better prognostic prediction. Similarly, combination of *EZH2* mutation and MD Anderson lower risk prognostic scoring system could identify 29% of lower risk MDS patients with a worse-than-expected prognosis^38^. However, a growing list of molecular alterations, such as RNA splicing genes and cohesin complex mutations, etc., were detected in MDS recently after these prior reports, and it is getting clear that some of these newly identified gene mutations have distinct phenotype and prognostic relevance^39^. In a mutational study of 111 genes in 738 patients with MDS or closely related neoplasm, Papaemmanuil et al.^11^ showed the total number of oncogenic mutations and cytogenetic lesions were negatively associated with LFS. Among them, eight mutations, including *CUX1*, *BCOR*, *RUNX1*, *IDH2*, *ASXL1*, *U2AF1*, *SRSF2*, and *TP53* mutations predicted shorter LFS while *SF3B1* mutation was associated with better LFS. Further, the numbers of driver mutations could further provide independent prognostic impact on LFS in patients with IPSS low and Int-1 risk.

Compared with IPSS, the IPSS-R incorporated more sophisticated parameters, including blasts percentages, cytogenetic classification and the depth of cytopenias to improve prognosis assessment for MDS patients^6^. IPSS-R has been the standard tool to risk stratify MDS patients. Haferlach et al.^12^ utilized a combination of conventional factors (age, gender, and IPSS-R) and mutations in 14 genes (*CBL*, *NARS*, *KRAS*, *ETV6*, *NPM1*, *LAMB4*, *NF1*, *PRPF8*, *RUNX1*, *TET2*, *ASXL1*, *EZH2*, *STAG2*, and *TP53*) as a novel prognostic model. It separated the patients into four risk groups^12^. In a study of Nazha et al.,^17^ incorporation of three mutations (*EZH2*, *SF3B1*, and *TP53*) into IPSS-R can improve the predictive power in 508 patients with primary and secondary MDS. Recently, Tefferi et al.^37^ showed that the survival impact of three adverse mutations, including *ASXL1*, *TP53*, and *SRSF2* mutations was most evident in IPSS-R very low- and low-risk patients, implying targeted sequencing may assist in decision-making in management of this group. However, the disease subtypes, adverse mutations identified and therapy choices were quite variable in these studies. More investigations are required to get clearer view. Further, the implication of mutations on clinical outcomes in Asian MDS patients may be different from that in western patients since disease natures of MDS and racial background are different between these two groups^18–20,32^. The current study was aimed to assess the additive prognostic impact of molecular profiling on IPSS-R and 2016 WHO classification in a large cohort of MDS patients in Asia. Further, an integrated risk-stratification model incorporating conventional risk factors and gene mutations was established to better risk-stratify MDS patients. We found LFS and OS were negatively associated with the number of mutations, compatible with the concept that clonal evolution with acquisition of genetic changes is associated with disease progression and poor survival^40^.

The association between specific cytogenetic change and particular mutation pattern, such as *TP53* with monosomal karyotype, *RUNX1* with trisomy 21 and *SF3B1* with inv^3^(q21q26.2) and del(11q) in MDS was recently reported, suggesting that mutation-induced genomic instability may result in specific cytogenetic abnormality^41^. Besides, the frequent co-existence of some mutations further provides the insights into molecular circuitry and mutual exclusiveness usually implies the functional redundancy in the development of MDS^11,39^. Most importantly, we showed that within each IPSS-R or 2016 WHO classification-defined risk group, two subgroups with different outcomes could be separated through mutational screening of five genes, including *CBL, IDH2, ASXL1*, *DNMT3A*, and *TP53*. Patients with these poor-risk mutations had an OS worse than others in the same IPSS-R or 2016 WHO subgroup, but similar to those in the next higher risk subgroup. These findings explain the clinical heterogeneity in the same IPSS-R risk groups. A substantial portion of patients in each IPSS-R risk group could be adjusted to different prognostic groups based on the integrated prognostic system; 22.9% of the IPSS-R very low- and low-risk patients could be redistributed into intermediate risk group, 31.5% of IPSS-R intermediate risk patients to high-risk group, and 53.8% of IPSS-R high-risk to very high-risk group. The incorporation of the status of these poor-risk mutations into the survival analysis would be especially helpful to identify patients with poorer prognosis for more aggressive treatment in the rather heterogeneous group of patients with lower risk MDS defined by conventional scoring systems.

The prognostic relevance of individual mutations in these five genes was largely comparable to those reported previously^8,12,42–44^. In contrast to the report of Haferlach et al.^12^, we could not find the independently prognostic relevance of *RUNX1* mutation, *TET2* mutation and *STAG2* mutation in our cohort although these three mutations were associated with poorer OS in univariate analysis, most likely owing to strong associations between these three mutations and older age and advanced IPSS-R^45,46^. On the other hand, *IDH2* and *DNMT3A* mutations were poor-risk genotypes in our cohort, but not in that of Haferlach et al^12^. Although, *SF3B1* mutation predicted better survival in the studies of Haferlach et al.^12^ and Nazha et al.^17^, *SF3B1* mutation was not a prognostic factor in our cohort. The possible explanation was *SF3B1* mutation was closely associated with older age and *DNMT3A* mutation, two independently poor prognostic markers. The different results obtained from these studies were probably due to differences in the patients recruited and in the treatment strategies. In the study of Haferlach et al.^12^, patients were relatively older (median 72.8 years) and 77.1% received supportive care; while in the study of Nazha et al.^17^, both primary and secondary MDS patients were recruited and only 20% received supportive care. Compared with these two reports, fewer cases were in IPSS-R very low- and low-risk groups in this study (27.7% vs. 54% and 54.5%). In contrast, about one half of patients had high or very high-risk MDS in our cohort.

The ultimate goal of management of MDS patients is to explore personalized therapy, thereby improve the treatment outcome while reduce the probability of leukemia transformation and treatment-related toxicity. Though most patients in this study only received palliative care, some did receive allogeneic HSCT, which gave us a chance to investigate the impact of genetic data on prognosis in patients undergoing this disease-modifying therapy. We previously showed that allogeneic HSCT could provide survival benefits for patients with IPSS-R high and very high risk^20^. However, the treatment choice for IPSS-R lower risk patients with documented poor-risk mutations, such as *CBL, IDH2, ASXL1*, *DNMT3A*, and *TP53* mutations is currently unclear. We showed that patients with unfavorable molecular genotypes had a better OS if they received allogeneic HSCT than those who did not. We also found allogeneic HSCT could significantly improve the survival of patients with IPSS-R lower risk, but with poor-risk mutations. It implied that HSCT might ameliorate the poor survival impact of the adverse-risk genotypes. Further prospective studies with more patients recruited are needed to verify this point.

The limitation in our study is that we also included patients with RAEBT and CMML defined by FAB classification. However, we distinctly showed that mutational characterization and integrated scoring system could well risk-stratify not only FAB-defined MDS patients, but also those defined by WHO classification. Second, with the limited number of patients receiving HMA treatment in our cohort, we could not determine whether HMA could provide survival benefits for these poor-risk patients.

In summary, early assessment of IPSS-R and mutational profiling of five relevant genes, including *CBL, IDH2, ASXL1*, *DNMT3A*, and *TP53*, may improve the prognostic stratification of MDS patients. Presence of these poor-risk mutations can also risk-stratify the patients independently of 2016 WHO classification. The clinically relevant integrated prognostic system further refines the prognostic prediction models and may guide the therapeutic decision. Supplementary information is available at Blood Cancer Journal’s website.

## Electronic supplementary material

Supplementary Tables and Figures
