# Supplementary material for: Incorporation of mutations in five genes in the revised International Prognostic Scoring System can improve risk stratification in the patients with myelodysplastic syndrome
Source: Blood Cancer J. 2018 Apr 4;8(4):39. doi: 10.1038/s41408-018-0074-7 (PMC5884776; doi:10.1038/s41408-018-0074-7)
Supplement: Supplementary file 1 — Supplementary Tables and Figures [file 41408_2018_74_MOESM1_ESM.docx]

**Supplementary Table 1**

**Clinical manifestation and laboratory features in 426 MDS patients**

| **Variables** | **Total Patients** |
| --- | --- |
| **Gender**^†^ |  |
| Male | 281 (66) |
| Female | 145 (34) |
| **Age (year)**^#^ | 67 (18-98) |
| **Lab data**^#^ |  |
| WBC (x10^9^/L) | 3,940 (440-355,300) |
| Hb (g/dL) | 8.3 (3.4-14.6) |
| Platelet (x10^9^/L) | 78 (2-931) |
| LDH (U/L) | 487 (145-6,807) |
| **FAB subtype**^††^ |  |
| RA | 155 (36.4) |
| RARS | 32 (7.5) |
| RAEB | 141 (33.1) |
| RAEB-T | 46 (10.8) |
| CMML | 52 (12.2) |
| **2008 WHO classification**^††^ |  |
| RCUD | 65 (15.3) |
| RCMD | 88 (20.7) |
| RARS | 32 (7.5) |
| RAEB1 | 68 (16.0) |
| RAEB2 | 73 (17.1) |
| MDS-U | 2 (0.4) |
| MDS with isolated del(5q) | 0 |
| AML, MDS | 46 (10.8) |
| MDS/MPN | 52 (12.2) |
| **2016 WHO classification**^††^ |  |
| MDS-SLD | 66 (20.1) |
| MDS-MLD | 83 (25.3) |
| MDS-RS-SLD | 20 (6.1) |
| MDS-RS-MLD | 14 (4.3) |
| MDS with isolated del(5q) | 2 (0.6) |
| MDS-EB-1 | 68 (20.7) |
| MDS-EB-2 | 73 (22.3) |
| MDS-U | 2 (0.6) |
| **IPSS**^†† §^ |  |
| Low | 70 (16.4) |
| INT-1 | 171 (40.1) |
| INT-2 | 111 (26.1) |
| High | 74 (17.4) |
| **IPSS-R**^††ζ^ |  |
| Very low | 12 (2.8) |
| Low | 106 (24.9) |
| Intermediate | 108 (25.3) |
| High | 106 (24.9) |
| Very high | 94 (22.1) |

^†^ Number of patients (% among the males or females)

^#^ Median (range)

^††^ number of patients (% among patients of each subgroup)

^§^ IPSS: Low, 0; intermediate (INT)-1, 0.5-1; INT-2, 1.5-2; and High, ≥ 2.5

^ζ^ IPSS-R: Very low, ≦1.5; Low, >1.5-3; intermediate, >3-4.5; High, >4.5-6; and Very high, >6

Abbreviations: CMML, chronic myelomonocytic leukemia; FAB, French-American-British classification; IPSS, international prognostic scoring system; IPSS-R, revised IPSS; MDS-EB1, MDS with excess blasts-1; MDS-EB2, MDS with excess blasts-2; MDS-SLD, MDS with single lineage dysplasia; MDS-MLD, MDS with multilineage dysplasia; MDS-RS-SLD, MDS with ring sideroblasts with single lineage dysplasia; MDS-RS-MLD, MDS with ring sideroblasts with multilineage dysplasia; MDS/MPN, myelodysplastic syndrome/myeloproliferative neoplasm; MDS-U, unclassified; RA, refractory anemia; RARS, refractory anemia with ring sideroblasts; RAEB, refractory anemia with excess blasts; RAEB-T, refractory anemia with excess blasts in transformation; RCUD, refractory cytopenia with unilineage dysplasia; RCMD, refractory cytopenia with multilineage dysplasia.

**Supplementary Table 2**

**Clinical manifestation and laboratory features in MDS patients with and without poor-risk mutations***

| **Variables** | **Total**  **(n=426)** | **Poor-risk Mutations*** **Absent**  **(n=248, 58.2%)** | **Poor-risk Mutations*** **Present**  **(n=178, 41.8%)** | **P value** |
| --- | --- | --- | --- | --- |
| **Gender**^†^ |  |  |  | 0.4571 |
| Male | 281 | 160 (56.9) | 121 (43.1) |  |
| Female | 145 | 88 (60.7) | 57 (39.3) |  |
| **Age (year)**^#^ | 67 (18-98) | 61.5 (18-98) | 70 (21-96) | <0.0001 |
| **Lab data**^#^ |  |  |  |  |
| WBC (x10^9^/L) | 3,940 (440-355,300) | 3490 (650-227,200) | 4960 (440-355,300) | <0.0001 |
| Hb (g/dL) | 8.3 (3.4-14.6) | 8.1 (3.4-14.6) | 8.5 (3.4-14.4) | 0.1642 |
| Platelet (x10^9^/L) | 78 (2-931) | 74 (2-721) | 84 (3-931) | 0.1504 |
| LDH (U/L) | 487 (145-6,807) | 466 (145-6,807) | 525 (225-3,756) | 0.1441 |
| **FAB subtype** |  |  |  | <0.0001 |
| RA | 155 | 128 | 27 |  |
| RARS | 32 | 21 | 11 |  |
| RAEB | 141 | 68 | 73 |  |
| RAEB-T | 46 | 17 | 29 |  |
| CMML | 52 | 14 | 38 |  |
| **2008 WHO classification** |  |  |  | <0.0001 |
| RCUD | 65 | 52 | 3 |  |
| RCMD | 88 | 75 | 13 |  |
| RARS | 32 | 21 | 11 |  |
| RAEB1 | 68 | 36 | 32 |  |
| RAEB2 | 73 | 32 | 41 |  |
| MDS-U | 2 | 1 | 1 |  |
| MDS with isolated del(5q) | 0 | 0 | 0 |  |
| AML, MDS | 46 | 17 | 29 |  |
| MDS/MPN | 52 | 14 | 38 |  |
| **2016 WHO classification** |  |  |  | <0.0001 |
| MDS-SLD | 66 | 55 | 11 |  |
| MDS-MLD | 83 | 68 | 15 |  |
| MDS-RS-SLD | 20 | 11 | 9 |  |
| MDS-RS-MLD | 14 | 12 | 2 |  |
| MDS with isolated del(5q) | 2 | 2 | 0 |  |
| MDS-EB-1 | 68 | 36 | 32 |  |
| MDS-EB-2 | 73 | 32 | 41 |  |
| MDS-U | 2 | 1 | 1 |  |
| **IPSS**^†† §^ |  |  |  | <0.0001 |
| Low | 70 | 45 | 25 |  |
| INT-1 | 171 | 125 | 46 |  |
| INT-2 | 111 | 53 | 58 |  |
| High | 74 | 25 | 49 |  |
| **IPSS-R**^ζ^ |  |  |  | <0.0001 |
| Very low | 12 | 10 | 2 |  |
| Low | 106 | 81 | 25 |  |
| Intermediate | 108 | 74 | 34 |  |
| High | 106 | 49 | 57 |  |
| Very high | 94 | 34 | 60 |  |

* Poor-risk mutations: *CBL, IDH2, ASXL1*, *DNMT3A*, and *TP53* mutations

^†^ Number of patients (% among the males or females)

^#^ Median (range)

^§^ IPSS: Low, 0; intermediate (INT)-1, 0.5-1; INT-2, 1.5-2; and High, ≥ 2.5

^ζ^ IPSS-R: Very low, ≦1.5; Low, >1.5-3; intermediate, >3-4.5; High, >4.5-6; and Very high, >6

Abbreviations: CMML, chronic myelomonocytic leukemia; FAB, French-American-British classification; IPSS, international prognostic scoring system; IPSS-R, revised IPSS; MDS-EB1, MDS with excess blasts-1; MDS-EB2, MDS with excess blasts-2; MDS-SLD, MDS with single lineage dysplasia; MDS-MLD, MDS with multilineage dysplasia; MDS-RS-SLD, MDS with ring sideroblasts with single lineage dysplasia; MDS-RS-MLD, MDS with ring sideroblasts with multilineage dysplasia; MDS/MPN, myelodysplastic syndrome/myeloproliferative neoplasm; MDS-U, unclassified; RA, refractory anemia; RARS, refractory anemia with ring sideroblasts; RAEB, refractory anemia with excess blasts; RAEB-T, refractory anemia with excess blasts in transformation; RCUD, refractory cytopenia with unilineage dysplasia; RCMD, refractory cytopenia with multilineage dysplasia.

**Supplementary Figure Legends**

**Supplementary Figure 1**

Number of gene mutations detected in patients with different MDS subtypes based on FAB (A), IPSS-R (B) and 2016 WHO classification (C)

**Supplementary Figure 2**

The diaphragm of the interaction of molecular genetic alterations and revised International Prognostic Scoring System (IPSS-R) classification in 426 MDS patients

**Supplementary Figure 3**

Frequency of mutations in 25 genes in 426 patients with different IPSS-R, which are shown in indicated colors.

**Supplementary Figure 4**

Frequency of gene mutations involved in common functional pathways stratified by different IPSS-R, which are shown in indicated colors.

**Supplementary Figure 5**

The Circos plots depicted the relative frequency and pairwise co-occurrence of genetic alterations in 426 MDS patients. The length of the arc corresponds to the frequency of the first gene mutation, and the width of the ribbon corresponds to the proportion of the second gene mutation.

**Supplementary Figure 6**

Kaplan–Meier survival curves for overall survival (A) and leukemia transformation rate (B) in 426 MDS patients stratified by IPSS-R

**Supplementary Figure 7**

Kaplan–Meier survival curves for overall survival (A) and leukemia-free survival (B) in 426 MDS patients stratified by the number of somatic mutations

**Supplementary Figure 8**

Kaplan–Meier survival curves for overall survival (A) and leukemia transformation rate (B) in 426 MDS patients stratified by presence of poor-risk mutations (*CBL, IDH2, ASXL1*, *DNMT3A*, and *TP53* mutations) or not

**Supplementary Figure 9**

Kaplan–Meier survival curves for overall survival (A) and leukemia transformation rate (B) in 328 MDS patients stratified by 2016 WHO classification

**Supplementary Figure 10**

Kaplan–Meier survival curves for overall survival among patients with poor-risk mutations (*CBL, IDH2, ASXL1*, *DNMT3A*, and *TP53* mutations) stratified by whether receiving allogeneic hematopoietic stem cell transplantation or not

**Supplementary Figure 1**

**(A)**

**(B)**

**(C)**

**Supplementary Figure 2**


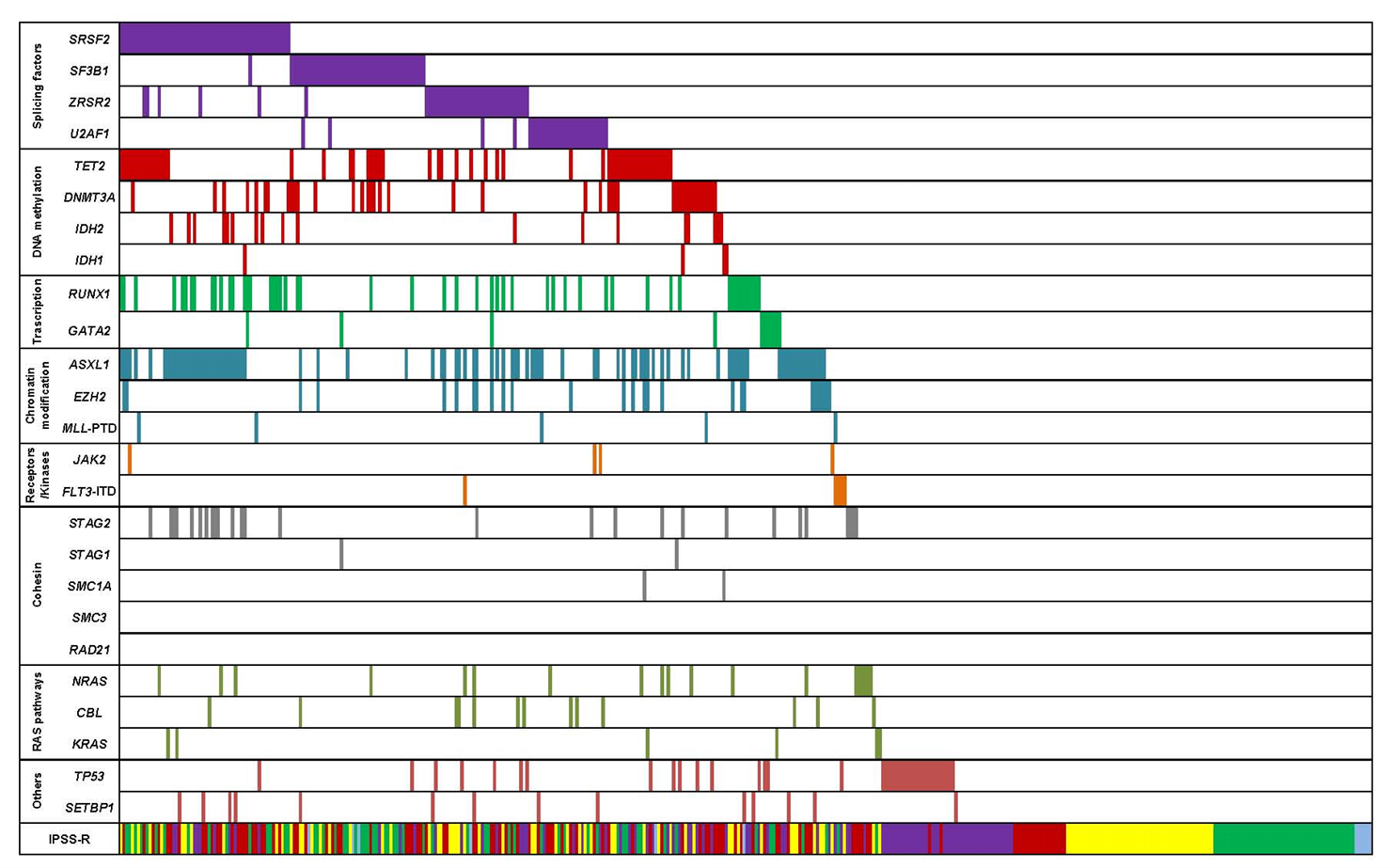

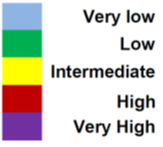


**Supplementary Figure 3**

**Supplementary Figure 4**

**Supplementary Figure 5**


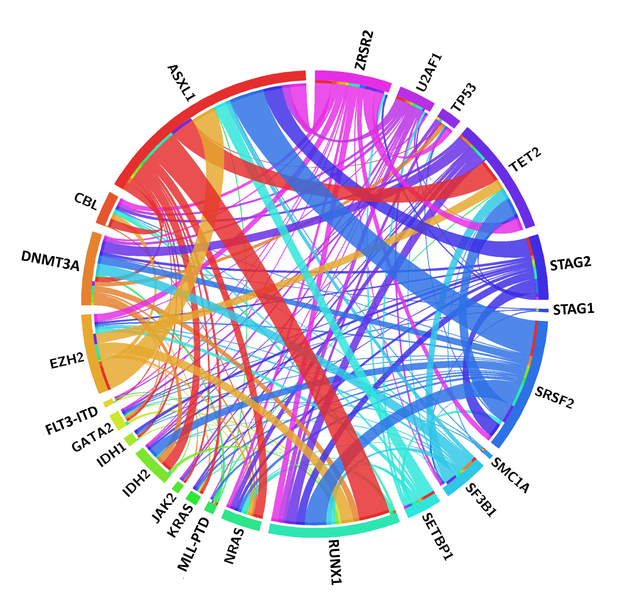


**Supplementary Figure 6**

**(6A)**


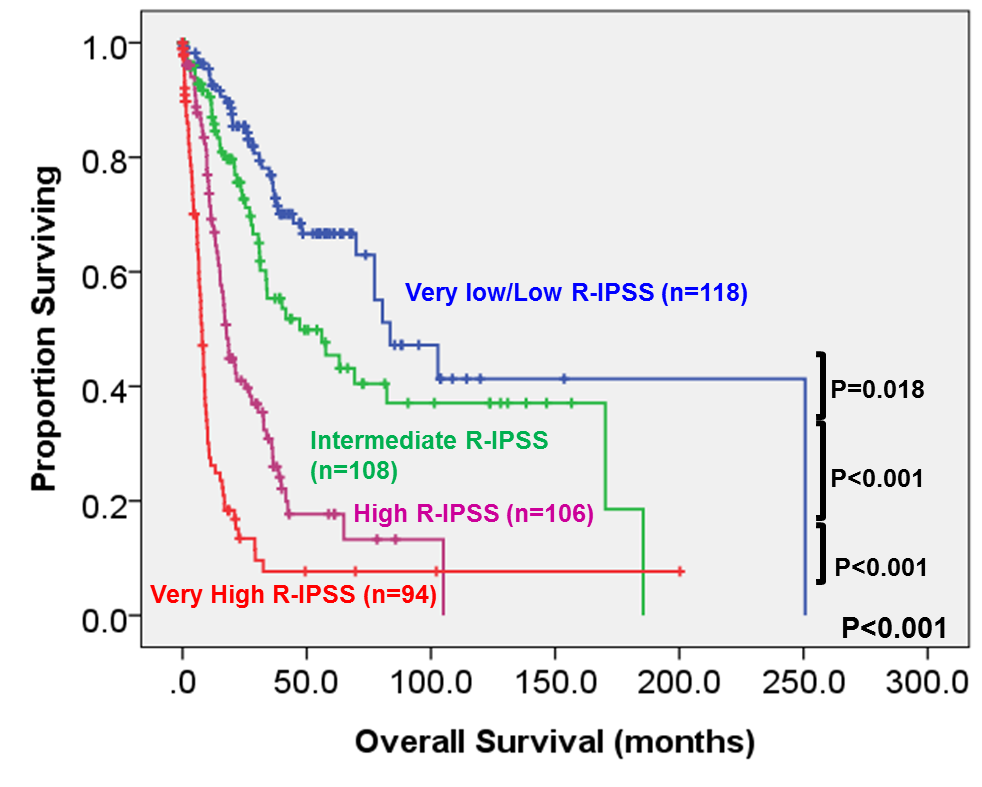


**(6B)**


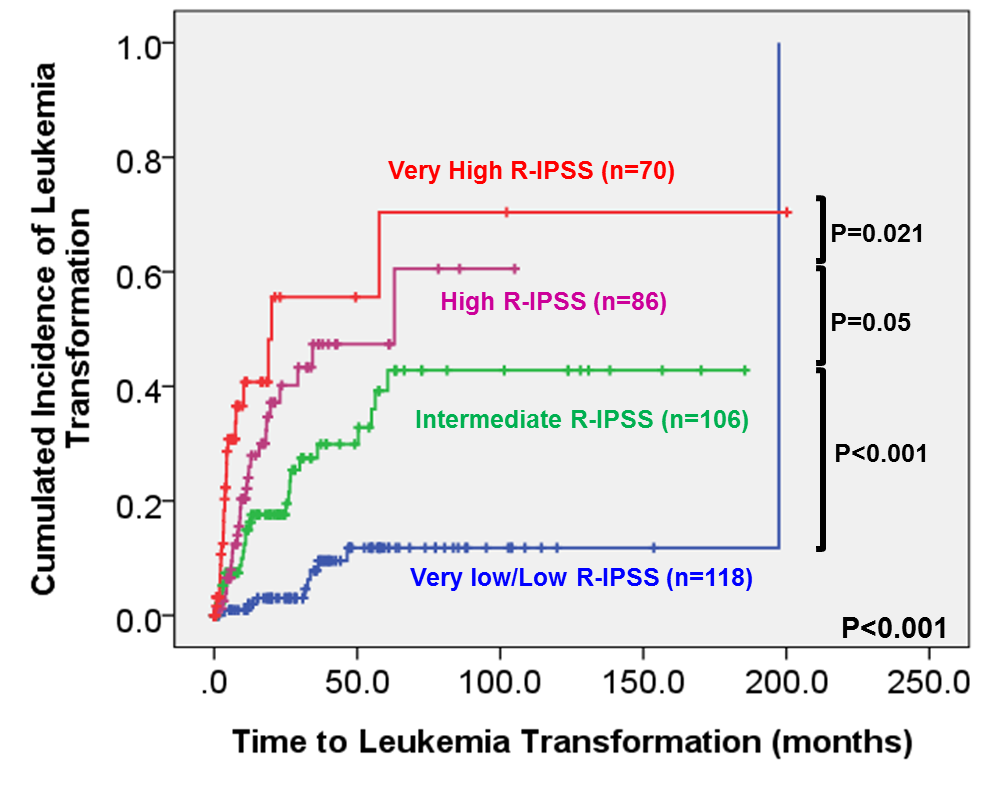


**Supplementary Figure 7**

**(7A)**


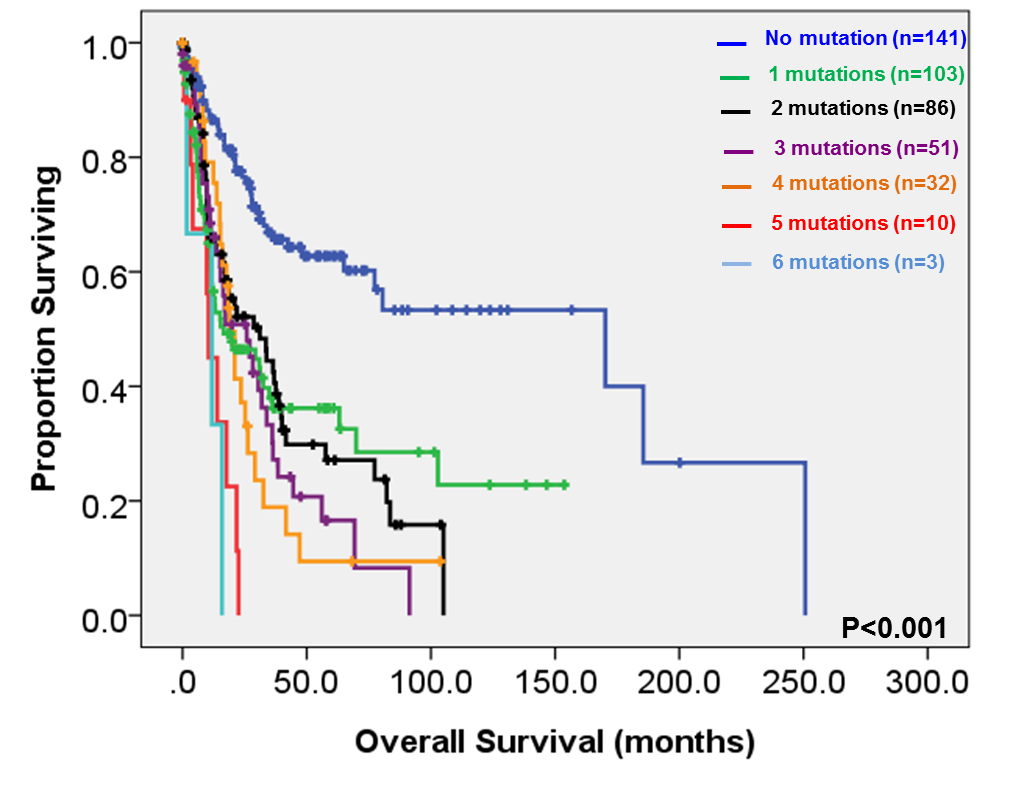


**(7B)**


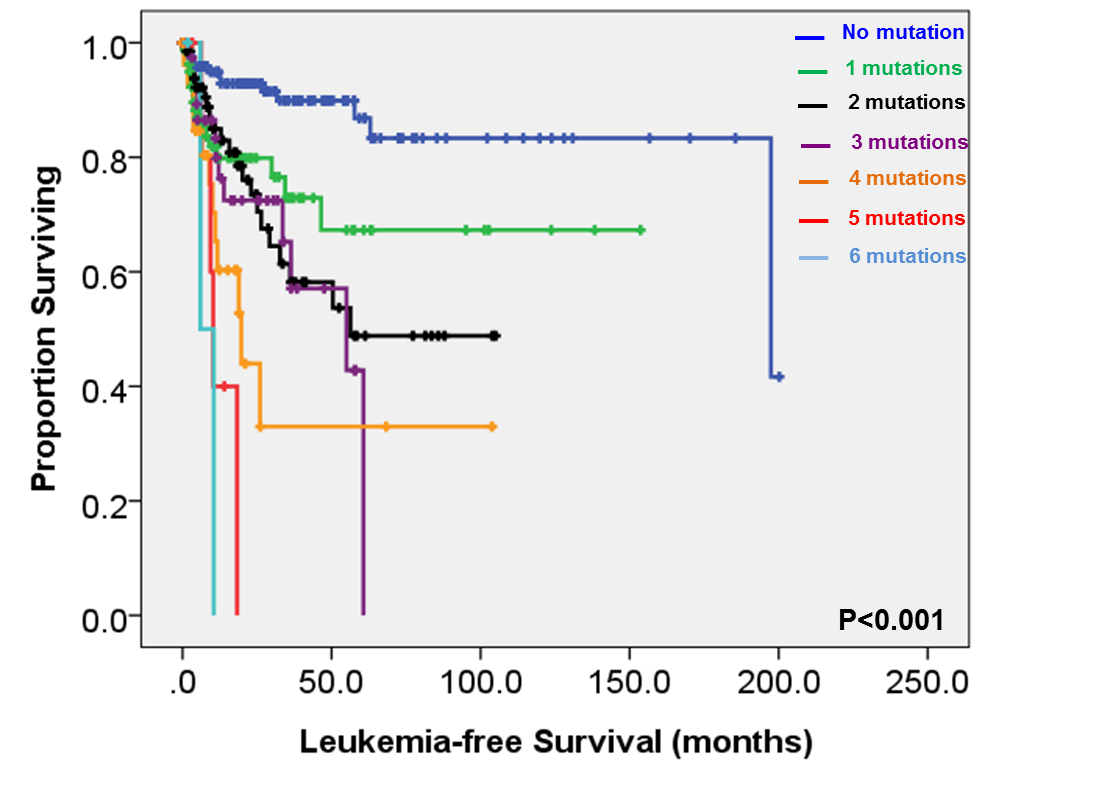


**Supplementary Figure 8**

**(8A)**

**
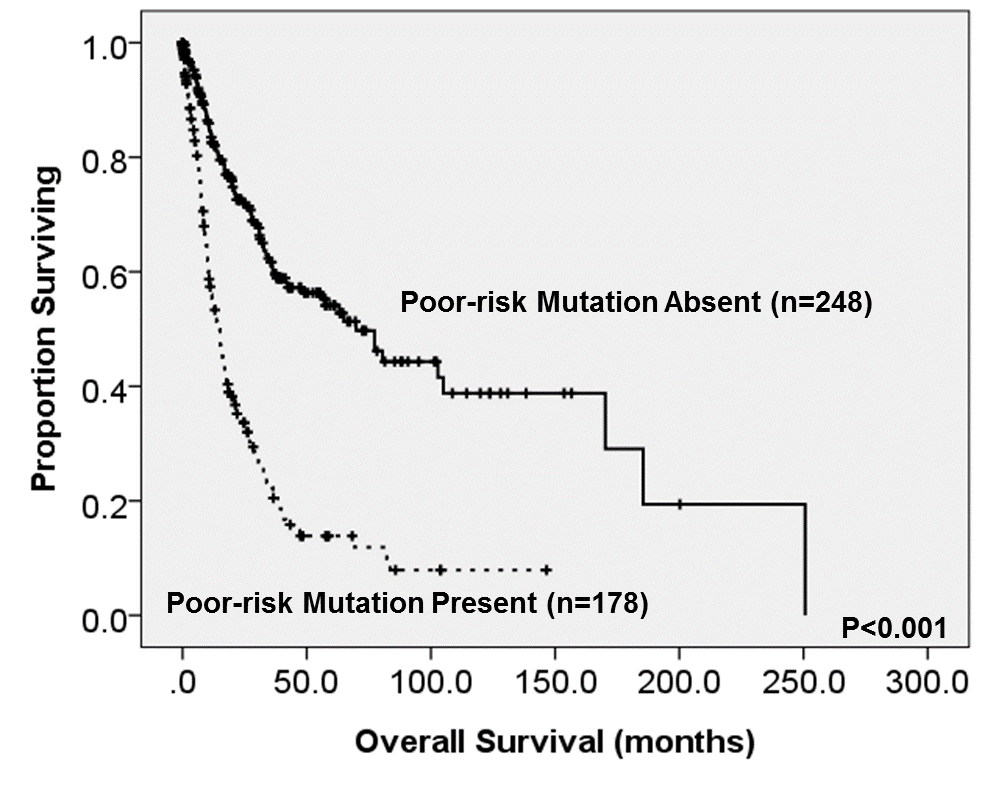
**

**(8B)**


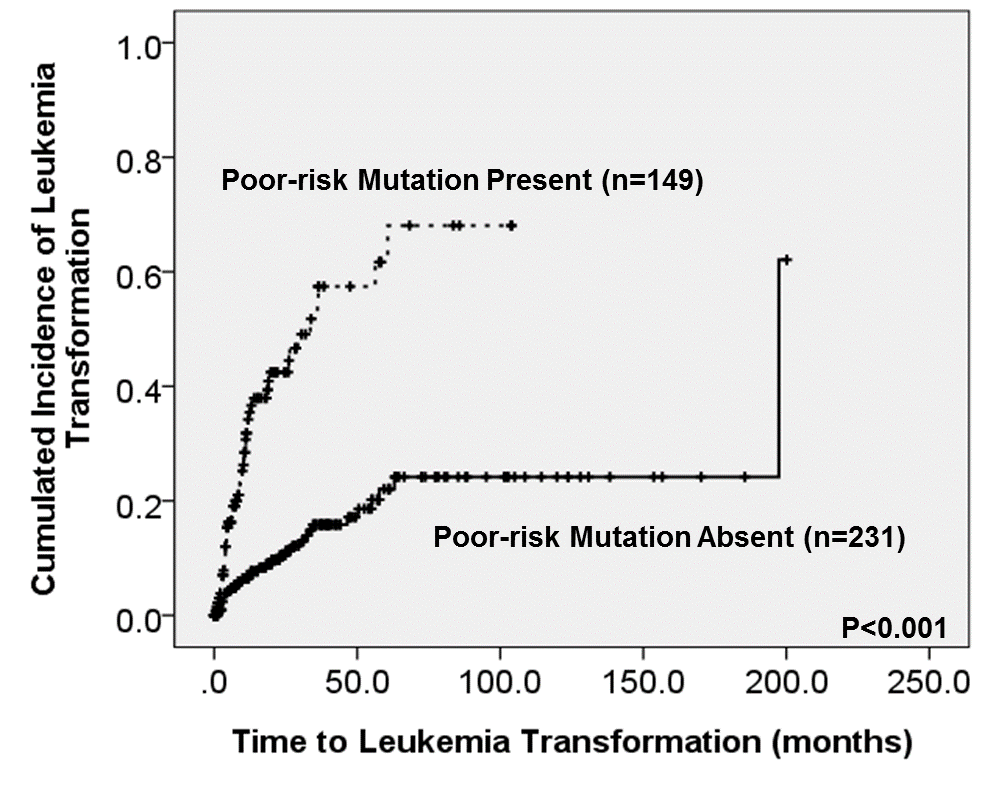


**Supplementary Figure 9**

**(9A)**


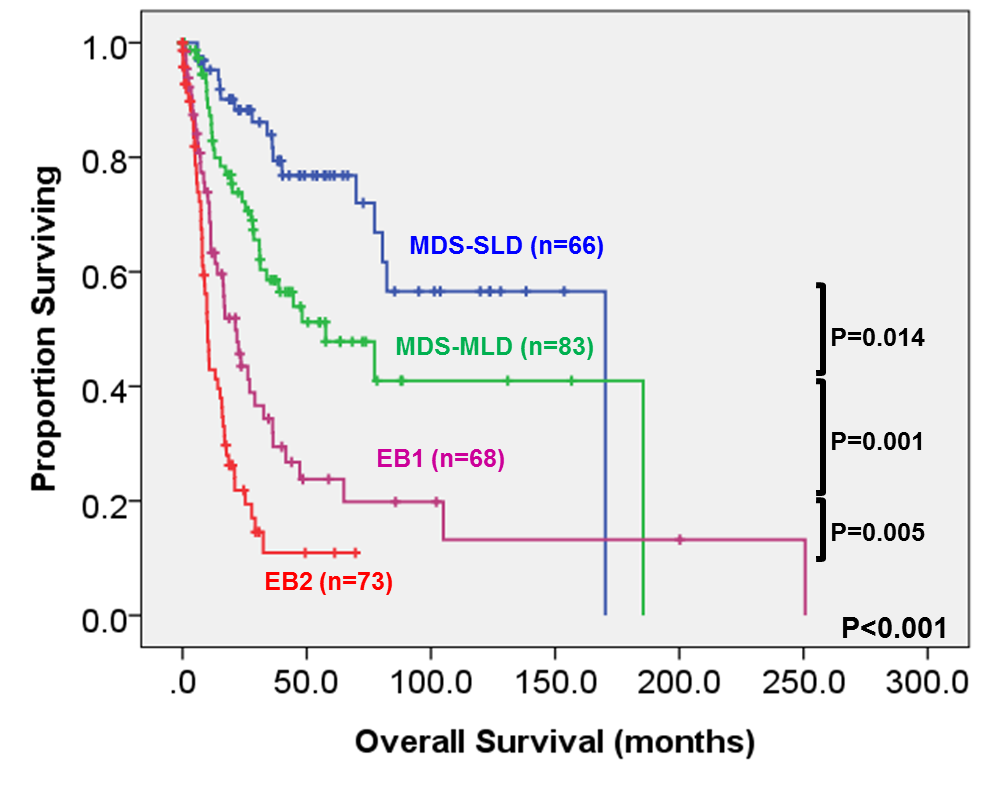


**(9B)**


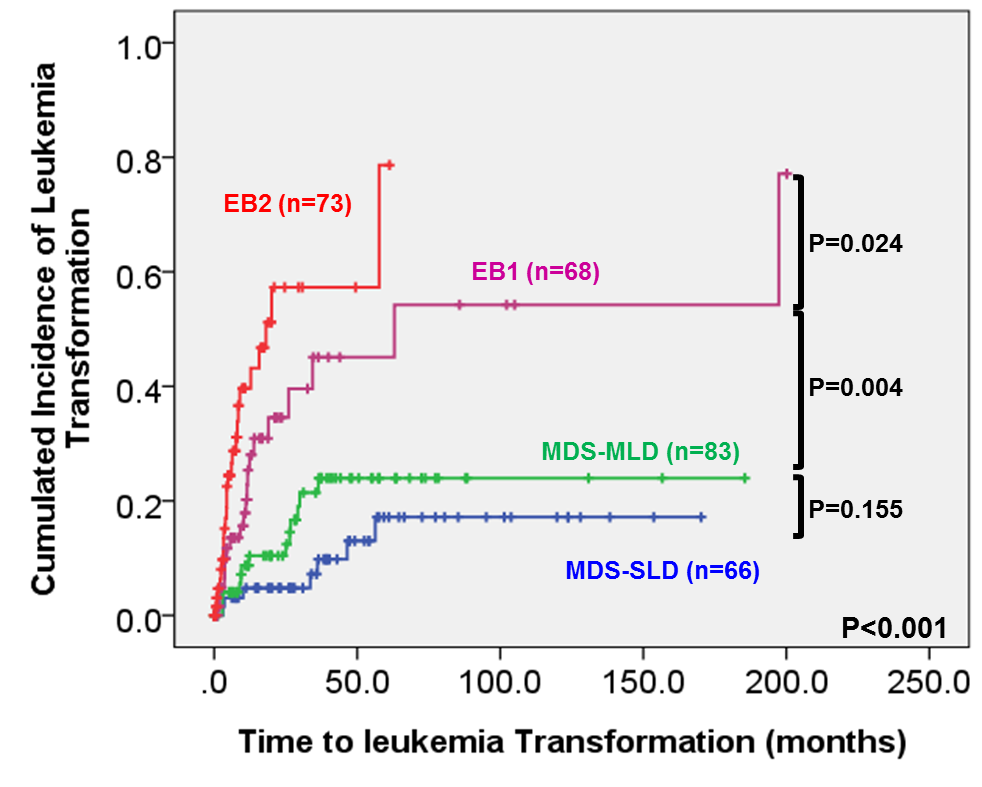


**Supplementary Figure 10
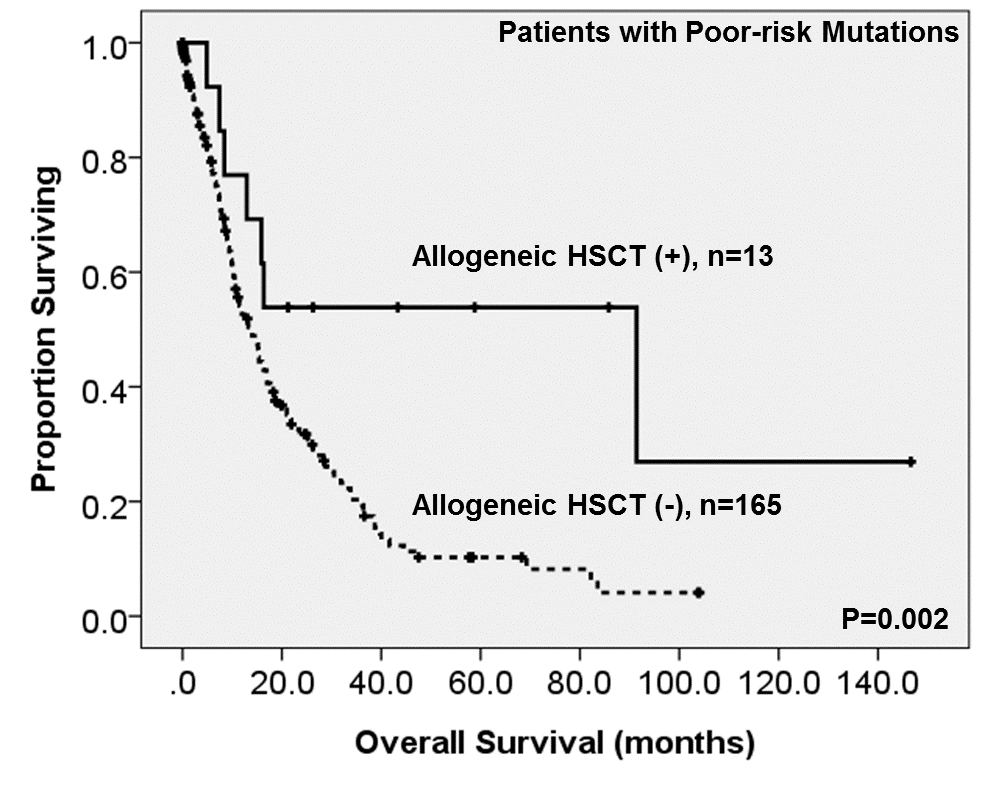
**
